# Prevalence of Human Papillomavirus Infection in the Female Partner of Infertile Couples Undergoing IVF/ICSI-ET and Subsequent Reproductive Outcomes

**DOI:** 10.3390/jcm11237185

**Published:** 2022-12-02

**Authors:** Sanhua Wei, Kaili Wang, Fang Cheng, Zhenhua Chang, Xiaoyan Ren, Zheng Liu, Mengxin Liu, Tao Yang, Xuhui Ma, Xiaojuan Xie, Xiaohong Wang

**Affiliations:** Reproductive Medicine Center, Department of Obstetrics and Gynaecology, Tang Du Hospital, The Air Force Military Medical University, Xi’an 710038, China

**Keywords:** human papillomavirus infection, assisted reproductive technology, embryonic development, pregnancy outcomes

## Abstract

We investigated the prevalence of human papillomavirus (HPV) infection in the female partner of infertile couples and the reproductive outcomes after in vitro fertilization/intracytoplasmic sperm injection-embryo transfer (IVF/ICSI-ET). We conducted a retrospective analysis on 8117 women from infertile couples who underwent IVF/ICSI treatment and evaluated the prevalence of HPV infection in these women. The prevalence of HPV infection in the female partner of infertile couples was 9.2% (747/8117). These HPV-infected female patients undergoing ART were divided into high-risk HPV (hrHPV) (*n* = 130) and low-risk HPV (lrHPV) groups (*n* = 94), and non-infected women patients formed the negative group (*n* = 126). Of the 747 cases infected with HPV, 529 showed hrHPV infection (70.82%; primarily genotypes 16, 52, 53, 58, and 59); 175 exhibited lrHPV infection (23.43%; primarily genotypes 6, 43, 44, 55, 61, and 81); and 43 cases were co-infected with hrHPV and lrHPV (5.76%). Except for the Day-3 high-quality embryo rate, there were no differences in ovum maturation, fertilization, implantation, clinical pregnancy, live birth, or miscarriage rates between women infected with HPV and non-infected women (*p* > 0.05); however, we noted an increased miscarriage rate after logistic regression analyses (OR, 0.16; 95% CI, 0.03–0.84; *p* = 0.041). For single-male-factor-induced infertility in couples (smHPV), although we likewise observed no differences in ovum maturation, fertilization, or implantation rates (*p* > 0.05) between the smHPV group and the negative group, we discerned diminutions in the Day-3 high-quality embryo rate (46.01% vs. 70.04%, *p* = 0.013), clinical pregnancy rate (46.67% vs. 57.94%, *p* = 0.003), and live birth rate (33.33% vs. 46.83%, *p* = 0.027) as well as an augmented miscarriage rate (11.11% vs. 4.76%, *p* = 0.003), respectively. Logistic regression analyses indicated that smHPV was a risk factor for decreased clinical pregnancy rate (OR, 4.17; 95% CI, 2.31–7.53; *p* < 0.001) and live birth rate (OR, 1.83; 95% CI, 0.81–2.14; *p* = 0.045) and elevated miscarriage rate (OR, 6.83; 95% CI, 2.22–21.00; *p* = 0.001). HPV infection in women was associated with increased miscarriage rate, and single-male-factor infertility influenced reproductive outcomes in couples undergoing IVF/ICSI treatment. Both were potentially due to HPV infection in the couple.

## 1. Introduction

It is acknowledged that sexually transmitted diseases (STDs) are a major cause of infertility, as 20–60% of cases of infertility in women are related to *Chlamydia trachomatis*, *Ureaplasma urealyticum*, and *Neisseria gonorrhoeae*, which cause cervical, tubal, and mucosal damage to the host [1,2]. Human papillomaviruses (HPV) are double-stranded DNA viruses that constitute the most common sexually transmitted causative agent infecting humans of reproductive age worldwide [3]. Among women of reproductive age, HPV infection is a potential risk factor that predisposes them to subsequent infertility [4], and it infects skin and mucosal and cutaneous epithelial cells [5]. HPV infection is highly correlated with precancerous and cancerous lesions of the cervix uteri, vulva, vagina, penis, and anogenital areas [3,6,7], and some studies indicate that HPV is detectable in cervical endometriotic and ovarian lesion tissues [8,9].

HPV infection is primarily self-limiting and can be cleared by self-immunity of the infected individual. However, persistent HPV infection can be carcinogenic and associated with precancerous lesions and cancer of the cervix and uterus in women and of the anogenital mucosa in women as well as men [10]. Persistent HPV infection has been linked to chronic inflammation [11], and infectious virion production may weaken the cells residing in the endometrium in association with infertility and miscarriage [12]. HPV infection increases the risk of spontaneous abortion as well as ectopic pregnancy, and different HPV genotypes may play disparate roles in adverse reproductive outcomes when using assisted reproductive technology (ART) [13].

While HPV infection may influence pregnancy outcome, this contention is controversial [14]; thus, the effects of HPV on women’s infertility and subsequent reproductive outcome require further study. Therefore, in the present study, we investigated the prevalence of HPV infection in women from infertile couples treated with IVF/ICSI-ET and assessed their reproductive outcomes.

## 2. Materials and Methods

### 2.1. Study Design

**Figure 1:**
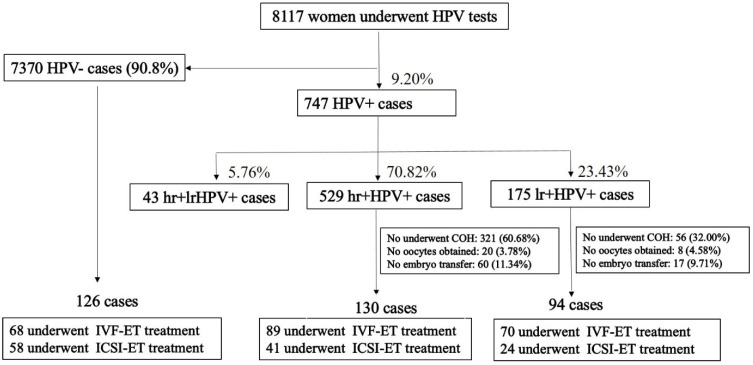
Study flowchart of HPV tests in women and IVF/ICSI-ET treatments. hrHPV, high-risk HPV; lrHPV, low-risk HPV; COH, controlled ovarian hyperstimulation; IVF, in vitro fertilization; ICSI, intracytoplasmic sperm injection; and ET, embryo transfer.

A total of 8117 women patients from infertile couples underwent HPV genotype testing; 747 patients were infected with HPV. The HPV (+) group was subsequently sorted into high-risk HPV infection (hrHPV+, 529/747, 70.82%), low-risk HPV infection (lrHPV+, 175/747, 24.42%), and high-risk and low-risk sub-groups (hrHPV+/lrHPV+, 43/747, 5.76%). Of 529 cases of hrHPV positivity and 175 cases lrHPV positivity, patients that did not undergo controlled ovarian hyperstimulation (COH), had no oocyte retrieved, and had no embryo transferred were excluded. Only 130 cases of hrHPV-positive and 94 cases of lrHPV-positive patients underwent IVF/ICSI-ET treatment. One hundred twenty-six HPV (−) patients were selected randomly and designated as negative control (Figure 1). We excluded all patients who showed an abnormal thin-prep cytologic test (TCT). Before the COH cycle, regular vaginal discharge and bacterial vaginitis (BV) were examined to exclude mycosis, trichomoniasis, *Gardnerella*, and *Neisseria gonorrhoeae*. Cervical swabs were examined to exclude *Chlamydia trachomatis, Ureaplasma urealyticum,* and *Mycoplasma genitalium*. Regular blood tests were also conducted to exclude HIV, HBV, HCV, and TP. All patients underwent a fresh-cycle embryo transfer or a frozen-embryo cycle embryo transfer after the COH cycle. The average transferred embryo number was 1.3 per cycle. We obtained detailed information on infertile patients that included age, years of infertility, body mass index (BMI), cause of infertility, baseline hormonal levels such as follicle-stimulating hormone (FSH) and anti-Müllerian hormone, and antral follicle count (AFC).

### 2.2. Determination of HPV Genotype

Sexual activities and vaginal medications were restricted prior to HPV analyses. Cervical discharges were swabbed for HPV detection, and genotyping was performed with a BioRad 100 Amplification and Luminex^®^ 200™ System (Thermo, Waltham, MA, USA) that detected 27 genotypes: high-risk genotypes 16, 18, 26, 31, 33, 35, 39, 45, 51, 52, 53, 56, 58, 59, 66, 68, and 82 as well as low-risk genotypes 6, 11, 40, 42, 43, 44, 55, 61, 81, and 83.

### 2.3. IVF/ICSI-ET Protocol

All patients underwent a standardized gonadotropin-releasing hormone (GnRH)-agonist long protocol or GnRH-antagonist protocol with oocyte retrieval, fertilization, and embryo transfer. For the GnRH-agonist long protocol, patients who underwent the IVF/ICSI-ET protocol experienced pituitary downregulation with a GnRH agonist administered at the midluteal phase. For the GnRH-antagonist protocol, patients initiated rFSH treatment on the second day of the cycle by once-daily injection. After five days of this treatment, we administered the antagonist cetrorelix acetate (Merck Serono, Darmstadt, Germany) daily, and the rFSH dose was adjusted according to the individual ovarian response as assessed by daily ultrasonographic examination. The antagonist treatment continued until the day of hCG injection. When at least two leading follicles reached 18 mm in diameter in the two COH protocols, ovulation was induced with recombinant α-HCG (5000 to 10,000 IU, Merck Serono, Darmstadt, Germany) and oocytes were collected between 36 and 38 h later. Oocytes were then fertilized by either conventional IVF or intracytoplasmic sperm injection (ICSI) [15], and all embryos were transferred on the third day after oocyte pickup with a standard ET protocol [16]. Vaginal progesterone (8% Crinone vaginal gel, Merck Serono, Darmstadt, Germany) was used daily from the day of embryo transfer (ET) to provide routine luteal support and maintain luteal function until the 10th week of pregnancy [17].

### 2.4. Embryonic Development and Reproductive Outcomes

Day-3 high-quality embryo rate, ovum maturation rate, fertilization rate, implantation rate, clinical pregnancy rate, live birth rate, and miscarriage rate were determined. Our rate calculations were as follows: ovum maturation rate = no. of D_0_ MII oocytes/no. of retrieved oocytes; fertilization rate = no. of Day-1 2PN embryos/no. of D_0_ MII oocytes; high-quality embryo rate = no. of Day-3 high-quality embryos/no. of Day-1 2PN oocytes; implantation rate = no. of implanted embryos (i.e., pregnancies)/transferred embryos; clinical pregnancy rate = pregnancy cycles/total cycles; and miscarriage rate = miscarriage cycles/total cycles.

### 2.5. Statistical Analysis

Measurements are presented as means ± standard deviation, and we applied the Statistical Package for the Social Sciences (SPSS, version 23.0, IBM, Armonk, NY, USA) for Windows for all statistical analyses. The Student’s *t* test and Chi-squared test were used to compare categorical variables, and a *p* value of <0.05 was considered to be statistically significant. We executed logistic regression analysis on reproductive outcomes, and odds ratios (ORs), 95% confidence intervals (CIs), and *p* values are reported.

## 3. Results

### 3.1. Prevalence of HPV Infection in Women from Infertile Couples

This was a retrospective study of women patients who had undergone IVF/ICSI-ET for infertility from January 1 to 31 December 2020 at Tangdu Hospital Reproductive Center, Xi’an, China. Of 8117 women of the infertile couples, we detected the DNA from at least one of the 17 hrHPV genotypes or 10 lrHPV genotypes in 9.20% (747/8117) of the total samples. Non-infected cases numbered 7370 (90.8%). hrHPV genotypes were detected in 70.82% (529/747) of HPV-positive patients, principally including genotypes 16, 52, 53, 56, 58, and 59. Genotypes 16 (116/747) and 52 (114/747) were the most common hrHPV infections; 175 cases (23.43%) involved lrHPV infection, primarily including genotypes 6, 43, 44, 55, 61, and 68. Genotype 61 was particularly prevalent (73/747). Forty-three cases of hrHPV- and lrHPV-mixed infections were detected, which constituted 5.76% of HPV-positive patients. For the hrHPV-positive group, 130 cases underwent IVF/ICSI-ET treatment, including 89 for IVF-ET and 41 for ICSI-ET. For the lrHPV-positive group, 94 cases underwent IVF/ICSI-ET treatment, including 70 for IVF-ET and 24 for ICSI-ET. HPV-negative cases (126) were selected as the random control group, which included 68 for IVF-ET and 58 for ICSI-ET (Figure 1).

All enrolled women patients were designated for a COH cycle and a fresh-embryos transfer or frozen-embryo transfer cycle. Statistical indicators included age, duration of infertility, body mass index (BMI), causes of infertility, levels of anti-Müllerian hormone (AMH) and follicle-stimulating hormone (FSH), AFC, no. of retrieved oocytes, no. of Day-0 M II oocytes, no. of Day-1 2PN zygotes, and no. of high-quality embryos per cycle.

### 3.2. Baseline Data on HPV-Infected Women from Infertile Couples

A total of 224 HPV-positive women (mean age, 32.3 ± 4.7 years) and 126 HPV-negative women (mean age, 31.9 ± 4.3 years) were enrolled in the present study (Figure 1 and Table 1). We compared the baseline characteristics of HPV-infected and non-HPV-infected patients who underwent COH and IVF/ICIS-ET treatment and noted no significant differences with respect to age (32.3 ± 4.7 years vs. 31.9 ± 4.3 years, *p* = 0.476), duration of infertility (4.2 ± 3.3 years vs. 3.6 ± 2.4 years, *p* = 0.123), or BMI (22.7 ± 3.0 vs. 23.1 ± 3.5, *p* = 0.282). However, women 26–40 years of age exhibited a higher infection rate (especially in women 26–35 years old) at above 70%, which may be associated with frequent sexual activity (Supplementary Table S1). There were no differences in baseline hormone levels for FSH or AMH or in AFC (*p* > 0.05), nor in the number of retrieved oocytes, the number of mature oocytes (Day-0 M II), or the number of fertilized oocytes (Day-1 2PN) per cycle between the HPV-positive and HPV-negative groups. However, HPV-infected women manifested a lower number of Day-3 high-quality embryos (4.4 ± 3.6 vs. 5.5 ± 3.1, respectively; *p* = 0.002) per cycle compared with non-HPV-infected women.

Fallopian-tube factor and pelvic factor were the main causes (nearly 50%) of infertility for both two groups, with 20% of infertility due to male factors, particularly oligozoospermia and asthenozoospermia (12.1%), in the HPV-infected group.

### 3.3. Embryonic Development and Pregnancy Outcomes in HPV-Positive and HPV-Negative Women

When we analyzed evaluation indicators of embryonic development and pregnancy outcomes, we observed no difference between the HPV-positive and -negative groups in ovum maturation rate (89.13% vs. 87.01%, *p* = 0.564) or fertilization rate (83.27% vs. 84.58%, *p* = 0.725), while the HPV-positive group had a lower Day-3 high-quality embryo rate (52.72% vs. 70.04%, *p* < 0.001, respectively). Regarding pregnancy outcomes, there were no significant differences between the HPV-positive and HPV-negative groups in the implantation rate (44.28% vs. 44.06%, *p* = 0.972), clinical pregnancy rate (55.36% vs. 57.94%, *p* = 0. 587), live birth rate (40.63% vs. 46.83%, *p* = 0.104), or miscarriage rate (6.25% vs. 4.76%, *p* = 0.556) (Table 2). The hrHPV- and lrHPV-infection groups did not differ in embryonic development or pregnancy outcome relative to the HPV-negative control group (*p* > 0.05) except for the Day-3 high-quality embryo rate (*p* < 0.001) (Supplementary Table S2).

To assess whether infection with HPV in women was associated with reproductive outcomes, we executed binary logistic regression analyses for HPV infection compared with uninfected status. Our results indicated that female infection with HPV was an independent risk factor for increased miscarriage rate (OR, 0.16; 95% CI, 0.03–0.84; *p* = 0.041). Women with HPV infection also showed a diminished clinical pregnancy rate (OR, 0.25; 95% CI, 0.08–0.77; *p* = 0.216) and live birth rate (OR, 0.31; 95%CI, 0.11–0.93; *p* = 0.437) in infertile couples. However, this difference was not significant (*p* > 0.05) (Table 3).

### 3.4. Embryonic Development and Pregnancy Outcomes in the smHPV and HPV-Negative Groups

Of the HPV-infected group, infertility in 45 infertile couples was caused by single male factors such as oligozoospermia and asthenozoospermia. Embryonic development and pregnancy outcomes of the single-male-factor group (defined as the smHPV group) were also evaluated, and we noted no difference in ovum maturation rate (88.20% vs. 87.01%, *p* = 0.418) or fertilization rate (83.94% vs. 84.58%, *p* = 0.217) between the smHPV group and the HPV-negative group. Implantation rate tended to be lower in the smHPV group relative to the HPV-negative group, but this was not significant (31.58% vs. 44.06%, *p* = 0.089). The smHPV group also exhibited a reduced Day-3 high-quality embryo rate (46.01% vs. 70.04%, *p* = 0.013), clinical pregnancy rate (46.67% vs. 57.94%, *p* = 0.003), live-birth rate (33.33% vs. 46.83%, *p* = 0.027), and increased miscarriage rate (11.11% vs. 4.76%, *p* = 0.003) compared with the HPV-negative group (Table 4). Our logistic regression analysis results indicated that single male factors comprised an independent risk for decreased clinical pregnancy rate (OR, 4.17; 95% CI, 2.31–7.53; *p* < 0.001), decreased live birth rate (OR, 1.83; 95% CI, 0.81–2.14; *p* = 0.045), and increased miscarriage rate (OR, 6.83; 95% CI, 2.22–21.00; *p* = 0.001) in infertile couples after infection of the female partner with HPV (Table 5).

## 4. Discussion

HPV infection can be spontaneously cleared within one to two years, but repeated infection is associated with multiple malignancies that include cervical, anogenital, and oropharyngeal cancers [18]. Over 200 different HPV genotypes have been identified [19], and the prevalence of HPV differs with respect to geographic location and socioeconomic status [14,20,21,22,23]. Several authors indicated that HPV prevalence was higher in pregnant women than in non-pregnant women and demonstrated overall HPV prevalence rates of 16.82% and 12.25%, respectively [24]. A case-control study suggested that HPV prevalence was 24.2% in pregnant women vs. 14.8% in non-pregnant women, and that HPV prevalence was age and genotype dependent [25]. In our study, HPV prevalence in women from infertile couples was 9.2%, and hrHPV was detected in 78.82% of all HPV-positive women. The predominant hrHPV genotypes were 16, 52, 53, 56, 58, and 59; and the predominant lrHPV genotypes were 6, 43, 44, 55, 61, and 68. Types 16 and 52 were the most common genotypes we observed in the infertile women, which is congruent with a recent report [25]. Genotype 61 is the predominant type in lrHPV, occupying 9.77% of the total HPV infection (73/747), which was higher than genotypes 6 and 11 (25/747). Our results also indicated that there was an elevated infection rate in women of infertile couples who were 26–40 years old (and particularly in women 26–35 years of age), accounting for 70% of total infections and potentially associated with frequent sexual activity in this age group.

As HPV DNA has not only been identified in the cervix but also in the placenta, fetal membranes, and amniotic fluid, pregnant women undergo an increased risk of HPV infection [18,24]. However, whether HPV infection exerts adverse effects on pregnancy outcomes remains controversial. While several researchers have suggested a higher HPV prevalence among women who suffered a spontaneous abortion in relation to normal pregnancies [26,27,28], others uncovered no correlation between HPV infection and the risk of spontaneous abortion, miscarriage, or preterm delivery [29,30]. In addition, attenuated HPV infection rates have been observed in patients with recurrent miscarriage, and it has been hypothesized that augmented immunoreactivity may be partially responsible for the recurrent pregnancy loss and that this may be protective against HPV infection [31]. Our results indicated that HPV infection in women of infertile couples did not alter ovum maturation or fertilization rate but reduced the Day-3 high-quality embryo rate (*p* < 0.001) regardless of whether the infection was either hrHPV or lrHPV. With respect to pregnancy outcomes, women’s HPV infection appeared to lower the clinical pregnancy and live birth rates and elevate the miscarriage rates (but not to a statistically significant extent). However, we noted after logistic regression analysis that women’s HPV infection increased the risk of miscarriage, which is the most common adverse pregnancy outcome [26,27,28,32,33,34].

HPV commonly infects both the male and female partners. Men can be infected with HPV in the penis, anus, and head and neck; and it can be detected in penile swabs and semen. There are reports of a significantly higher HPV infection in infertile couples compared to the general population (20.9% vs. 8.2%) [35], and HPV also affects semen parameters [36]. One study indicated a statistically significant correlation between the rate of pregnancy loss and positivity for HPV DNA in the male partner of infertile couples compared with non-infected couples (66.7% vs. 15%, respectively) [32]. Pregnancy rate was reduced and the miscarriage rate was increased after HPV infection in both women and men [33]. Depuydt et al. reported that the pregnancy rate with intra-uterine insemination (IUI) declined when the sperm DNA fragmentation index (DFI) exceeded 26%; and sperm samples containing HPV exhibited a significantly higher DFI compared with HPV-negative sperm samples (29.8% vs. 20.9%, respectively; *p* = 0.011) [37]. However, Hana et al. recently reported that men with hrHPV-positive semen samples showed altered seminal parameters that included lower semen volume, sperm concentration, and total sperm count relative to men with HPV-negative samples; but there was no association between seminal hrHPV infection and pregnancy outcomes that included spontaneous abortion [36]. Thus, the impact of HPV on male fertility and associated reproductive outcomes remains debatable.

In our study, infertility in 45 couples was caused by single male factors that mainly included oligozoospermia and asthenozoospermia, and this was possibly associated with HPV infection. The single-male-factor group (smHPV group) manifested a lower Day-3 high-quality embryo rate, clinical pregnancy rate, and live birth rate, and it had an increased miscarriage rate compared with the HPV-negative group. Logistic regression analysis indicated that single male factors comprised an independent risk for decreased clinical pregnancy rate, decreased live birth rate, and increased miscarriage rate in infertile couples in which the female partner was infected with HPV. Our results suggested that HPV infection could cause semen parameters to change, which is possibly one of the reasons for oligozoospermia and asthenozoospermia, and that it would contribute to lower high-quality embryo rate [38], reduced clinical pregnancy and live birth rates and increased the miscarriage rate. We did not investigate the prevalence of male-partner HPV infection and changes in semen parameters, so further investigation of male-partner infection status is necessary.

Several studies indicated that HPV infection was associated with spontaneous preterm birth (sPTB), defined as delivery between 28 and 37 weeks of gestation [14]. In normal pregnancy, 17.5% of HPV infection occurs at the cervix, which is significantly lower than in sPTB patients, and cervical cytology shows that HPV infection generates placental abnormalities and preterm birth [39,40]. Hr-HPV infection was also associated with a risk of premature rupture of the membranes [41], and persistent HPV-16/18 infection was related to an increased risk of preterm birth independent of cervical treatment [42]. In our study, we observed no rise in the sPTB rate but did observe an attenuation in the live birth rate between the HPV-infected and non-HPV-infected groups.

This study also has some limitations. First, we only investigated the prevalence of human papillomavirus infection in the female partner of infertile couples before IVF/ICSI-ET treatment. We have no repeated HPV DNA detection results with intervals three to six months beginning at IVF/ICSI-ET treatment, which could have confirmed ongoing HPV infection. Second, in our study, HPV infection in males were not tested despite the fact that previous reports have shown a correlation between HPV+ male and pregnancy loss [32,33]. Finally, the number of HPV-infected cases undergoing IVF/ICSI-ET was limited, and the influence HPV infection on reproductive outcomes in females or both couples needs to be clarified by further large population-based studies.

In summary, our results indicated that HPV-infected women of infertile couples did not show alterations in ovum maturation, fertilization, implantation, clinical pregnancy, live birth, or miscarriage rates regardless of hrHPV or lrHPV infection, but they did exhibit lower high-quality embryo rates with HPV infection. HPV infection in these women was associated with a reduced miscarriage rate, and single-male-factor-induced infertility influenced reproductive outcomes of couples undergoing IVF/ICSI treatment. However, simultaneous determination of HPV status for both female and male partners of infertile couples is required to further clarify this phenomenon.

## Figures and Tables

**Table 1:** Characteristics of infertile women patients who underwent IVF/ICSI-ET treatment.

Characteristics	Positive		Positive (*n* = 224)	Negative (*n* = 126)	*p* Value
	hrHPV (*n* = 130)	lrHPV (*n* = 94)			
Age (years)	31.7 ± 4.3	33.7 ± 5.1	32.3 ± 4.7	31.9 ± 4.3	0.476
Duration of infertility (years)	3.9 ± 2.8	4.6 ± 4.0	4.2 ± 3.3	3.6 ± 2.4	0.123
BMI (kg/m^2^)	22.8 ± 3.0	22.6 ± 3.1	22.7 ± 3.0	23.1 ± 3.5	0.282
Causes of infertility, no. (%)					
Female factors					
Fallopian tube factor	41 (31.5)	38 (40.4)	79 (35.3)	46 (36.5)	
Pelvic factor	16 (12.3)	10 (10.6)	26 (11.6)	14 (11.1)	
Endometriosis	0 (4.3)	4 (4.3)	4 (1.8)	2 (1.6)	
Ovulatory dysfunction	5 (3.8)	2 (2.1)	7 (3.1)	4 (3.2)	
PCOS	2 (1.5)	0 (0)	2 (0.9)	4 (3.2)	
Mixed female factors	24 (18.5)	2 (2.1)	26 (11.6)	15 (11.9)	
Other female factors	8 (6.2)	11 (11.7)	19 (8.5)	2 (1.6)	
Male factors					
Oligozoospermia/asthenozoospermia	16 (12.3)	11 (11.7)	27 (12.1)	16 (12.6)	
Other male factors	11 (8.5)	7 (7.4)	18 (8.0)	17 (13.5)	
Mixed female and male factors	2 (1.5)	4 (4.3)	6 (2.7)	2 (1.6)	
Unexplained infertility	5 (3.8)	5 (5.3)	10 (4.5)	4 (3.2)	
Baseline hormone concentrations					
FSH (IU/L)	6.6 ± 3.0	7.2 ± 3.1	6.9 ± 3.0	6.5 ± 2.2	0.244
AMH (ng/mL)	3.0 ± 1.9	2.6 ± 1.6	2.9 ± 1.8	2.8 ± 2.0	0.888
AFC (L)	7.0 ± 3.6	6.3 ± 3.8	6.7 ± 3.7	6.1 ± 3.5	0.176
AFC (R)	7.0 ± 3.5	6.2 ± 3.2	6.7 ± 3.4	6.6 ± 3.2	0.864
No. of retrieved oocytes	11.6 ± 5.6	10.6 ± 3.7	11.2 ± 4.9	11.2 ± 5.1	0.902
No. of Day-0 MII oocytes	10.2 ± 5.2	9.6 ± 3.7	10.0 ± 4.6	9.8 ± 4.3	0.723
No. of Day-1 2PN oocytes	8.4 ± 4.4	8.1 ± 3.6	8.3 ± 4.0	8.3 ± 3.7	0.963
No. of Day-3 high-quality embryos per cycle	4.2 ± 4.0	4.6 ± 2.9	4.4 ± 3.6	5.5 ± 3.1	0.002 **

Notes: BMI, body mass index; PCOS, polycystic ovary syndrome; oligozoospermia, sperm concentration ≤15 × 10^6^/mL or total sperm count ≤ 39 × 10^6^; asthenozoospermia, sperm progressive motility ≤ 30%; FSH, follicle-stimulating hormone; and AMH, anti-Müllerian hormone. Mixed female factors, mixed with two or more female infertility factors; mixed women and male factors, mixed with two or more factors of infertility for both sexes. All Day-3 high-quality embryos were developed from two pronuclear zygotes and met the following criteria: (1) more than five blastomeres; (2) a blastomere size difference of less than 20%; and (3) fragmentation of less than 50%. ** *p* < 0.01.

**Table 2:** Embryonic development and pregnancy outcomes in HPV-positive group and HPV-negative group.

Embryonic Development and Pregnancy Outcomes	HPV Positive (*n* = 224)	HPV Negative (*n* = 126)	*p* Value
Ovum maturation rate ^a^	89.13% (2230/2502)	87.01% (1232/1416)	0.564
Fertilization rate ^b^	83.27% (1857/2230)	84.58% (1042/1232)	0.725
High-quality embryo rate ^c^	52.72% (978/1855)	70.04% (699/998)	<0.001 **
Implantation rate ^d^	44.28% (151/341)	44.06% (89/202)	0.972
Clinical pregnancy rate ^e^	55.36% (124/224)	57.94% (73/126)	0.587
Live birth rate ^f^	40.63% (91/224)	46.83% (59/126)	0.104
Miscarriage rate ^g^	6.25% (14/224)	4.76% (6/126)	0.156

Notes: ^a^ Ovum maturation rate was defined as the no. of Day-0 MII oocytes per cycle/the no. of retrieved oocytes per cycle. ^b^ Fertilization rate was defined as the no. of Day-1 2PN embryos per cycle/the no. of Day-0 MII oocytes per cycle. ^c^ The high-quality embryo rate was defined as the no. of Day-3 high-quality embryos per cycle/the no. of Day-1 2PN embryos per cycle. ^d^ Implantation rate was defined as the no. of implanted embryos (i.e., pregnancies) per cycle/the no. of transferred embryos per cycle. ^e^ Clinical pregnancy rate was defined as clinical pregnancy cycles/total cycles. ^f^ Miscarriage rate was defined as miscarriage cycles/total cycles. ^g^ Live birth rate was defined as live birth cycles/total cycles. ** *p* < 0.01.

**Table 3:** Logistic regression analyses for reproductive outcomes between HPV-positive group and HPV-negative group.

Variable	Clinical Pregnancy Rate		Miscarriage Rate		Live-Birth Rate	
	OR (95% CI)	*p* Value	OR (95% CI)	*p* Value	OR (95% CI)	*p* Value
HPV-positive vs. HPV-negative	0.25 (0.08–0.77)	0.216	0.16 (0.03–0.84)	0.041 *	0.31 (0.11–0.93)	0.437

Notes: Factors were adjusted for age, duration of infertility, BMI, causes of infertility, baseline hormone levels, and the number of high-quality embryos per cycle. OR, odds ratio; CI, confidence interval. * *p* < 0.05.

**Table 4:** Embryonic development and pregnancy outcomes for single-male-factor HPV-positive and HPV-negative groups.

Embryonic Development and Pregnancy Outcomes	smHPV-Positive (*n* = 45)	HPV-Negative (*n* = 126)	*p* Value
Ovum maturation rate	88.2% (523/593)	87.01% (1232/1416)	0.418
Fertilization rate	83.94% (439/523)	84.58% (1042/1232)	0.217
High-quality embryo rate	46.01% (202/439)	70.04% (699/998)	0.013 *
Implantation rate	31.58% (23/69)	44.06% (89/202)	0.089
Clinical pregnancy rate	46.67% (21/45)	57.94% (73/126)	0.003 **
Live birth rate	33.33% (15/45)	46.83% (59/126)	0.027 *
Miscarriage rate	11.11% (5/45)	4.76% (6/126)	0.003 **

Notes: smHPV, single-male-factor for infertility. * *p* < 0.05, ** *p* < 0.01.

**Table 5:** Logistic regression analyses of reproductive outcomes between single-male-factor HPV-positive and HPV-negative groups.

Variable	Clinical Pregnancy Rate		Miscarriage Rate		Live Birth Rate	
	OR (95% CI)	*p* Value	OR (95% CI)	*p* Value	OR (95% CI)	*p* Value
smHPV-positive vs. HPV-negative	4.17 (2.31–7.53)	<0.001 **	6.83 (2.22–21.00)	0.001 **	1.83 (0.81–2.14)	0.045 *

Notes: Factors were adjusted for age, duration of infertility, BMI, causes of infertility, baseline hormone levels, and the number of high-quality embryos per cycle. OR, odds ratio; CI, confidence interval. * *p* < 0.05, ** *p* < 0.01.

## Data Availability

The datasets used and analyzed during the current study are available from the corresponding author on reasonable request.

## Supplementary Materials

The following supporting information can be downloaded at: https://www.mdpi.com/article/10.3390/jcm11237185/s1, Table S1: Age distribution of female HPV-positive patients; Table S2: Embryonic development and pregnancy outcomes in hrHPV-positive group, lrHPV-positive group, and HPV-negative group.
