# Supplementary material for: Prevalence of Human Papillomavirus Infection in the Female Partner of Infertile Couples Undergoing IVF/ICSI-ET and Subsequent Reproductive Outcomes
Source: J Clin Med. 2022 Dec 2;11(23):7185. doi: 10.3390/jcm11237185 (PMC9741338; doi:10.3390/jcm11237185)
Supplement: Supplementary file 1 [file jcm-11-07185-s001.zip › jcm-2001145-supplementary.pdf]

Journal of
Clinical Medicine

Supplementary Table S1. Age distribution of female HPV-positive patients.

Age (Years)	hrHPV	lrHPV	hr + lrHPV
	n = 529	*n* = 175	*n* = 43
<25	26	6	2
26–30	238	59	14
31–35	172	63	23
36–40	65	36	1
41–45	20	9	3
>45	8	2	0

Notes: hrHPV, high-risk HPV infection; lrHPV, low-risk HPV infection.

Supplementary Table S2. Embryonic development and pregnancy outcomes in hrHPV-positive group, lrHPV-positive group, and HPV-negative group.

Embryonic Development and Pregnancy Outcomes	HPV Positive (*n* = 224)		HPV Negative (*n* = 126)	*p* Value		
	hrHPV (*n* = 130)	lrHPV (*n* = 94)		hrHPV/lrHPV	hrHPV/HPV Negative	lrHPV/HPV Negative
Ovum maturation rate	88.26% (1331/1508)	90.44% (899/994)	87.01% (1232/1416)	0.393	0.909	0.294
Fertilization rate	82.42% (1097/1331)	84.54% (760/899)	84.58% (1042/1232)	0.858	0.718	0.692
High-quality embryo rate	49.95% (548/1097)	57.34% (430/758)	70.04% (699/998)	0.099	<0.001 **	<0.001 **
Implantation rate	46.52% (87/187)	41.56% (64/154)	44.06% (89/202)	0.285	0.789	0.389
Clinical pregnancy rate	53.08% (69/130)	56.38% (53/94)	57.94% (73/126)	0.367	0.291	0.781
Live birth rate	39.23% (51/130)	42.55% (40/94)	46.83% (59/126)	0.119	0.119	0.214
Miscarriage rate	6.92% (9/130)	5.32% (5/94)	4.76% (6/126)	0.667	0.501	0.497

Notes: hrHPV, high-risk HPV infection; lrHPV low-risk HPV infection. ** $p < 0.01$.
